# Assessment of body composition, sarcopenia and protein intake in mild to moderate Parkinson’s disease

**DOI:** 10.3389/fnut.2025.1507545

**Published:** 2025-07-07

**Authors:** Danielle Pessoa Lima, Pauliana Alencar Monteiro, João Rafael Gomes de Luna, Antonio Brazil Viana-Júnior, Lucas Tadeu Rocha Santos, Samuel Brito de Almeida, Rayanne Rego Ferreira Saldanha, Madeleine Sales de Alencar, Fábia Karine de Moura Lopes, Átila Pereira Alencar, Raisa Carvalho de Brito Arcanjo Chaves, Wallena Cavalcante Brito, Paulo Ribeiro Nóbrega, Alexandre Bastos Lima, Pedro Braga-Neto, Jarbas de Sá Roriz-Filho, Renan Magalhaes Montenegro Júnior

**Affiliations:** ^1^Division of Geriatrics, Department of Clinical Medicine, Federal University of Ceará, Fortaleza, Brazil; ^2^Medical School, University of Fortaleza, Fortaleza, Brazil; ^3^Postgraduate Program in Public Health, Medical School, Federal University of Ceará, Fortaleza, Brazil; ^4^Clinical Research Unit of Walter Cantídio Universitary Hospital, Federal University of Ceará, Fortaleza, Brazil; ^5^Division of Neurology, Department of Clinical Medicine, Federal University of Ceará, Fortaleza, Brazil; ^6^Center of Health Sciences, State University of Ceará, Fortaleza, Brazil

**Keywords:** body composition, sarcopenia, protein intake, nutritional status, Parkinson’s disease

## Abstract

Parkinson’s disease (PD) is a progressive neurodegenerative disorder characterized by motor and non motor symptoms. Nutritional status, particularly protein intake, plays a crucial role in managing PD symptoms and preventing complications such as sarcopenia. In Brazil, only 38% of the elderly frequently consume protein-rich foods. The aim of this study was to evaluate the association of protein quantity in the diet of patients with mild to moderate PD with clinical, physical, and body composition factors. A cross-sectional study was conducted involving PD patients in Hoehn and Yahr (HY) stages 1 to 3. Protein intake was assessed using dietary recall, body composition was measured using dual-energy X-ray absorptiometry (DXA), and sarcopenia was assessed following the Revised European Consensus of Sarcopenia. The mean SARC-F score was 3.97, with 51% patients screening positive for sarcopenia. The average handgrip strength was 29, 20% patients had low handgrip strength. The average Short Physical Performance Battery (SPPB) score was 8.87. Confirmed sarcopenia was present in 10% of the sample. Low protein intake (<1 g/kg/day) was observed in 35% of patients and was associated with positive screening of sarcopenia (SARC-F ≥ 4), low lean appendicular mass, and high fat mass index. We did not include patients with severe disease who exhibit more malnutrition, dysphagia, cognitive impairment, dyskinesias, and consequently more sarcopenia. We cannot, therefore, extrapolate these results to all patients with PD. Accordingly, a deeper understanding of the relationship between protein intake and body composition in PD may enhance long-term outcomes for patients.

## 1 Introduction

Parkinson’s disease (PD) is a progressive neurodegenerative disease with both motor (bradykinesia, rigidity, tremor and postural instability) and non-motor symptoms, including gastroparesis, constipation, depression, anxiety, and cognitive impairment in addition to dopaminergic medication side effects, which can make it difficult to maintain proper nutrition (1). Besides, levodopa absorption is impaired by amino acids in the small intestine. Spacing meals and redistributing proteins to allow a gap of 1 hour between levodopa administration and eating enhances the drug’s bioavailability. This non-pharmacological approach is often used to improve levodopa brain levels (2).

PD is the most rapidly increasing neurological disorder globally, with a 60% rise in age standardized prevalence between 1990 and 2021. With an aging population, the impact of PD and the resulting strain on health and social care systems are expected to rise, making this increase in prevalence a public health issue. Currently, PD is the second most prevalent neurodegenerative disorder worldwide. While the global prevalence of PD has increased over recent decades, there are significant geographic variations in this trend, with a particularly higher increase observed in countries such as China and the United States (3).

A systematic review and meta-analysis identified a continuous increase in PD prevalence from 1980 to 2023, with a more pronounced acceleration between 2004 and 2023. This growth is associated with various risk factors beyond population aging, including environmental and metabolic influences, lifestyle factors, and dietary habits, all of which are impacted by industrialization and urbanization. Environmental exposures, such as air pollution, pesticides, solvents, and heavy metals, are more prevalent in countries with higher socioeconomic indices, including the Sociodemographic Index (SDI) and Human Development Index (HDI), and may contribute to the rising prevalence of PD in these regions (3).

This discrepancy suggests that environmental factors may play a crucial role in the rising incidence of PD. In response to this growing concern, the World Health Organization (WHO) has recommended reducing exposure to specific environmental factors associated with PD development, with particular emphasis on pesticides, trichloroethylene, and air pollution (4).

Sarcopenia is characterized by a loss of muscle mass and strength and is associated with unfavorable outcomes such as falls, frailty, loss of physical function, loss of independence and poorer quality of life (5). Although recognized as a muscle disease since 2016, the diagnosis of sarcopenia is rarely made or documented in medical records (5, 6). Decreased caloric intake may result in a decline in muscle mass and quality (7). Nutrition is a significant contributing factor in the intricate causes of sarcopenia and frailty (8). In Brazil, according to the Household Budget Survey published in 2020, only 38% of the elderly frequently consume protein-rich foods (9). Additionally, the prevalence of low protein intake reaches 21.5, 46.7%, or 70.8% when the adopted cut-off point is 0.8 g/kg/day, 1.0 g/kg/day, or 1.2 g/kg/day, respectively (10).

Sarcopenia is more prevalent in patients with PD due to multiple factors, including mitochondrial dysfunction, chronic inflammation, an imbalance between protein synthesis and degradation, and reduced physical activity (11, 12). Studies indicate that the prevalence of sarcopenia among individuals with PD ranges from 10.9 to 31.4%, depending on the diagnostic criteria applied (7). Furthermore, the coexistence of sarcopenia and PD is associated with a reduced quality of life, an increased risk of falls, and accelerated mobility decline (5, 7). Despite these findings, the role of dietary protein intake in sarcopenia-related outcomes in PD remains insufficiently understood. Investigating this relationship is essential for the development of targeted nutritional strategies aimed at mitigating sarcopenia and its associated impairments in individuals with PD. Adequate protein ingestion may affect the net balance of muscle protein production (13, 14).

An adequate protein diet may reverse or at least delay functional decline in frail older persons. There is a scarcity of evidence on protein intake in PD (15). The aim of this study was to evaluate the association of protein quantity in the diet of patients with mild to moderate PD with clinical, physical, and body composition factors.

## 2 Materials and methods

### 2.1 Study design

**Figure 1:**
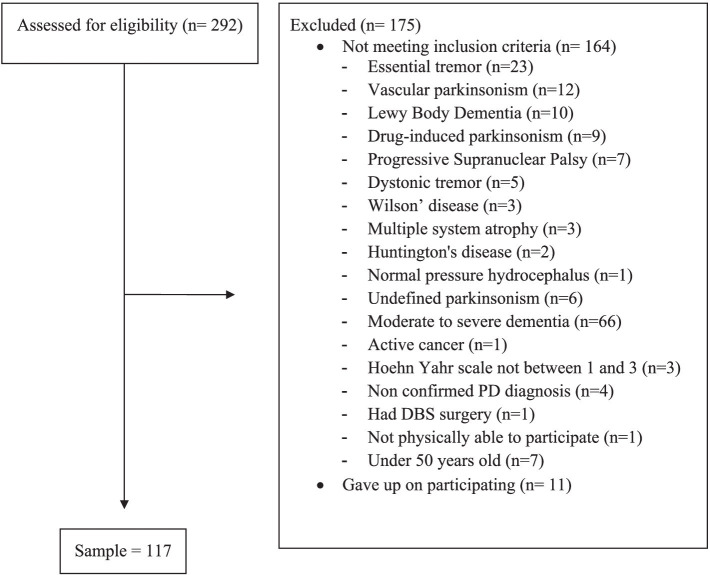
Flowchart of the recruitment process.

This study was carried out from May 2021 to April 2022 in the Neurology outpatient clinic at a public tertiary Brazilian hospital. The clinical diagnosis of PD and classification in stages 1 to 3 on the modified Hoehn & Yahr scale were eligibility criteria, as well as having the ability to stand and walk without assistance and being aged 50 years or older. Figure 1 shows the flowchart of the recruitment process of the study.

### 2.2 Elegibility criteria

We excluded patients with severe health conditions or uncontrolled chronic disease that could compromise their safety in carrying out the research procedures or the interpretation of findings, such as: heart failure functional class III and IV of the New York Heart Association; chronic kidney disease on dialysis; neurological diseases with motor impairment (except PD); moderate to severe dementia (Clinical Dementia Rating 2 and 3); severe chronic obstructive pulmonary disease (dyspnea with minor efforts) or very severe (dyspnea at rest and/or oxygen therapy); diagnosis of cancer, except localized prostate cancer and localized skin cancer, and patients with conditions that would complicate the interpretation of the dual-energy x-ray absorptiometry (DEXA) (use of oral contrast or radio-nuclides in the last 72 h; pregnancy; deep brain stimulation; cardiac pacemaker).

### 2.3 Medical and neurological evaluation

All patient evaluations and examinations were carried out 1 to 3 h after the patients had taken their antiparkinsonian medication (ON phase) and followed their normal medication schedules throughout the data collection phase of the study.

We performed a general medical assessment (demographic data, symptoms/complaints, comorbidities, medications in use), assessed depressive symptoms through the Geriatric Depression Scale-15 (GDS-15) and cognitive status using the Mini Mental Status Exam (MMSE). We also evaluated the number of falls in the last year. Clinical assessment of PD was performed evaluating symptoms/complaints, HY staging, the motor parkinsonian symptoms through the Movement Disorders Society-Unified Parkinsonian Disease Severity-part III (UPDRS-III), levodopa equivalent dose (LED) and capacity of daily living by Schwab and England (SE).

### 2.4 Sarcopenia assessment

We followed the Revised European Sarcopenia Consensus (EWGSOP2) to investigate sarcopenia. We performed the sarcopenia screening tool called SARC-F, anthropometric measurements (right calf, weight, height), muscular strength assessment (handgrip, using a SAEHAN dynamometer) and physical performance through the Short Physical Performance Battery (SPPB). The SARC-F questionnaire includes five questions that assess hand strength for lifting a 5 kg weight, thigh strength for standing up from a chair, strength and balance for walking between rooms and climbing 10 flights of stairs, and a history of falls (5).

### 2.5 Body composition analysis

We evaluated body composition using the DEXA Lunar Prodigy Advance (General Electric Healthcare) software enCORE version 17. Whole-body DEXA scans were obtained using the manufacturer’s recommendations. The following parameters were analyzed: fat mass (kg), lean mass (kg), percentage of total body fat (%BF), fat mass index (FMI, kg/m^2^), volume of visceral adipose tissue (VAT), percentage of fat by body segments, and relative skeletal muscle index (RSMI). We interpreted the FMI (total mass fat/height^2^) according to the National Health and Nutrition Survey (NHANES III) cutoff points (16), considering a normal range for women of 5 to 9 kg/m^2^ and for men of 3 at 6 kg/m^2^.

### 2.6 Dietary assessment

We assessed protein intake using the retrospective dietary survey, 24-h dietary recall. We calculated protein and calcium measurements per kilogram of weight per day from the 24-h dietary recall using Dietbox® Nutrition Software. We considered a protein intake of 1 g/kg/day or more as the minimum adequate for good muscle health in this study (17).

### 2.7 Definitions

We used the following definition of LED: the dose that produces the same level of symptomatic control as 100 mg of immediate-release L-dopa, according to the systematic review of Tomlinson et al. (18). A fall was considered a situation in which the patient involuntarily fell to the floor or another lower level and was not due to a convulsive event, vehicle or bicycle accident or syncope. The Calf Circumference (CC) measurement was taken using a non-stretchable tape measure, with the patient seated and the knee bent at 90°, feet spaced 20 cm apart, at the maximum circumference in the plane perpendicular to the longitudinal line of the calf. The patient had to remove his/her clothing on the lower body to measure the CC on bare skin. Subcutaneous tissues were not compressed (19).

We followed the recommendations of the EWGSOP2 regarding the measurement of manual handgrip strength and cutoff points (<27 kg for men and <16 kg for women) (5). We used the Southampton protocol to measure the handgrip strength (the patient seated with forearms supported on the arms of the chair, wrist just above the end of the chair arm, in a neutral position, thumb pointing upwards, feet flat on the floor, three attempts on each side, alternating sides, with the highest score from all six attempts recorded) (20).

The SPPB was developed to assess physical performance. The test includes measures of standing balance, 4-meter gait speed, and the time it takes to get up from a chair five times. Patients were told to keep their balance by standing with their feet together before spending 10 s in each of the semi-tandem and tandem postures, which involve positioning one foot’s heel near to the other foot’s big toe. Participants were told to walk along an 8-meter track (with 2-meters of acceleration and 2-meters of deceleration) at their normal pace to assess their gait speed with a stopwatch. Participants were taught to stand up and sit down five times as fast as they could with their arms crossed over their chests to assess their ability to get out of a chair. This was not done until individuals demonstrated that they could stand up once without using their arms. The overall SPPB score was calculated, with a score of less than 8 indicating inadequate physical performance and a maximum score of 12 (21).

Probable sarcopenia was defined as low handgrip strength. Confirmed sarcopenia was diagnosed according to EWGSOP 2 as follows: low muscle strength and low muscle mass—according to the relative skeletal muscle index (RSMI) obtained through DEXA < 7 kg/m^2^ for men and < 5.5 kg/m^2^ for women (5). The RSMI is obtained by the appendicular lean mass adjusted for height squared in meters. Low muscle mass is defined as a decrease in appendicular muscle mass two standard deviations below the mean for young healthy adults (5).

### 2.8 Ethical considerations

All individuals involved in the study gave their written consent after being fully informed about the research, which was approved by the Research Ethics Committee of Hospital Universitário Walter Cantidio (registration number 91075318.1.0000.5045). The study’s researchers spoke with and assessed each patient.

### 2.9 Statistical analysis

Descriptive statistics were presented as numbers (percentage) for categorical variables and as mean ± standard deviation (median) for quantitative variables. Bivariate analysis for probable and confirmed sarcopenia were performed using the Pearson’s chi-squared test and Fisher’s exact test for categorial variables. Spearman correlation coefficients were calculated to verify the association between protein intake and markers of interest. The Mann–Whitney U test was used to assess quantitative independent variables since they were not normally distributed except for calf circumference, for which Student’s T-test was used. Variables with *p* < 0.05 entered logistic regression to identify those independently associated with protein intake <=1 g/kg/d. Statistical analyses were performed using RStudio 2023.03.0.

## 3 Results

Table 1 presents the clinical characteristics of the sample. The study included 117 patients, with 48 (41%) being women. The average age of the participants was 66 years (± 11 years). The most common comorbidities were hypertension (*n* = 52, 44%), depression (*n* = 34, 29%), dyslipidemia (*n* = 18, 15%), and type 2 diabetes mellitus (*n* = 15, 13%). The average protein intake was 1.19 ± 0.41 g/kg/day, and the average calcium intake was 666 ± 348 mg/day. Low protein intake was observed in 35% (*n* = 41) of the patients. On average, patients were taking 5.3 ± 2.29 medications, with antidepressants and antihypertensives being the most used.

**Table 1:** Clinical and parkinsonian features of the sample.

Variables	*N* = 117^1^
Clinical variables	
Sex	
Female	48 (41%)
Male	69 (59%)
Age	66 ± 11 (67)
Hypertension	52 (44%)
Diabetes	15 (13%)
Dyslipidemia	18 (15%)
Vertebral Osteoarthritis	9 (7.7%)
Knee Osteoarthritis	9 (7.7%)
Constipation	52 (44%)
Calcium intake	666 ± 348 (654)
Protein intake	1.19 ± 0.41 (1.17)
Protein intake	
Reduced	41 (35%)
Normal	76 (65%)
Low appetite	17 (15%)
Actual Smoker	3 (2.6%)
Actual mild to moderate alcohol user	16 (14%)
Depression	
Yes	34 (29%)
No	83 (71%)
Number of medicines	5.30 ± 2.29 (5.00)
Benzodiazepine use	12 (10%)
Antidepressants use	43 (37%)
Antihypertensives use	39 (33%)
Typical antipsychotics use	0 (0%)
Atypical antipsychotics use	4 (3.4%)
Anticonvulsivant use	9 (7.7%)
Anticholinesterase use	5 (4.3%)
Antidiabetic use	9 (7.7%)
Measurements related to PD	
Schwab-England	85 ± 11 (90)
Hoehn Yahr	
1–2	28 (24%)
2.5–3	89 (76%)
UPDRS Part III score	43 ± 15 (41)
UPDRS 3.9 Standing from a chair	1.63 ± 0.75 (2.00)
UPDRS 3.10 Gait	2.50 ± 0.61 (3.00)
UPDRS 3.12 Postural stability	2.43 ± 1.12 (2.00)
UPDRS 3.13 Posture	2.66 ± 1.07 (2.00)
PIGD	9.22 ± 2.57 (9.00)
Dyskinesia	
Present	59 (50%)
Absent	58 (50%)
Visual hallucinations	
Present	23 (20%)
Absent	94 (80%)
Disease duration	10 ± 6 (9)
Levodopa equivalent dose	735 ± 334 (750)
GDS	5.01 ± 3.42 (4.00)
MMSE	24.1 ± 4.1 (25.0)

PD, Parkinson Disease; UPDRS, Unified Parkinson’s Disease Rating Scale; PIGD, Postural Instability and Gait Disorder; GDS, Geriatric Depression Scale; MMSE, Mini Mental State Examination.

1 *n* (%); Average ± Standard deviation (Median).

Regarding PD characteristics, 28 (24%) patients were in the early stages of the disease (HY 1–2), while 89 (76%) were in the moderate stages (HY 2.5–3). The average disease duration was 10 ± 6 years, with a mean levodopa equivalent dose of 735 ± 334 mg/day. The average UPDRS part 3 score was 43 ± 15. Approximately half of the patients (*n* = 59) experienced dyskinesias, and 23 (20%) reported visual hallucinations.

The mean SARC-F score was 3.97 ± 2.74, with 59 (51%) patients screening positive for sarcopenia. The average handgrip strength was 29 ± 11 kg, and 23 (20%) patients had low handgrip strength. The average SPPB score was 8.87 ± 2.60. Regarding body composition, the average BMI was 26.2 ± 4.4 kg/m^2^, the average calf circumference was 33.5 ± 3.6 cm, and the average RSMI was 7.27 ± 1.23 kg/m^2^. Confirmed sarcopenia was present in 10% (*n* = 12) of the sample.

Table 2 details physical performance and body composition results. The average number of falls in the past 6 months was 3.85 ± 18.76, and the average walking speed was 1.38 ± 0.52 m/s.

**Table 2:** Physical performance and body composition features of the sample.

Variables	*N* = 117^1^
Measurements related to physical performance	
Number of falls in the last 6 months	3.85 ± 18.76 (0.00)
Gait speed	1.38 ± 0.52 (1.40)
SARC-F score	3.97 ± 2.74 (4.00)
Positive sarcopenia screening	59 (51%)
Handgrip strength	29 ± 11 (28)
Low handgrip strength	23 (20%)
Total SPPB score	8.87 ± 2.60 (9.00)
Physical activity at least 3x/week for 30 min	
Yes	38 (75%)
No	13 (25%)
One or more falls in last 6 months	46 (39%)
Two or more falls in last 6 months	30 (26%)
Measurements related to body composition	
Appendicular lean mass	18.6 ± 4.7 (18.0)
Total lean mass	43 ± 9 (43)
Confirmed sarcopenia	12 (10%)
RSMI	7.27 ± 1.23 (7.14)
Low RSMI	23 (20%)
FMI Classification	8.5 ± 3.5 (8.3)
High	71 (61%)
Low	6 (5.2%)
Normal	39 (34%)
BMI	26.2 ± 4.4 (26.5)
Low BMI	18 (15%)
VAT volume	920 ± 708 (703)
Arms fat %	31 ± 10 (29)
Legs fat %	31 ± 10 (31)
Trunk fat %	33 ± 11 (35)
Android fat %	35 ± 13 (37)
Gynoid fat %	34 ± 11 (35)
Osteoporosis WHO Criteria	
Osteoporosis	36 (31%)
Osteopenia	51 (44%)
Normal	29 (25%)
Calf circumference	33.5 ± 3.6 (33.5)
Low calf circumference	26 (23%)

SARC-F, Simple questionnaire to rapidly diagnose sarcopenia; SPPB, Short Physical Performance Battery; RSMI, Relative Skeletal Muscle Index; FMI, Fat Mass Index; BMI, Body Mass Index; VAT, Visceral Adipose Tissue; WHO, World Health Organization.

1 *n* (%); Average ± Standard deviation (Median).

Table 3 shows the bivariate analysis results comparing clinical variables between patients with low and normal protein intake. Low protein intake was significantly associated with female gender, lower calcium intake and decreased appetite.

**Table 3:** Bivariate analysis of clinical variables and protein intake.

Variables	Protein intake		*p* value^2^
	Low, *N* = 41^1^	Normal, *N* = 76^1^	
Clinical variables			
Sex			**<0.001**
Female	28 (68%)	20 (26%)	
Male	13 (32%)	56 (74%)	
Age	66 ± 11 (66)	66 ± 10 (67)	0.706
Hypertension	16 (39%)	36 (47%)	0.386
Diabetes	3 (7.3%)	12 (16%)	0.191
Dyslipidemia	8 (20%)	10 (13%)	0.363
Vertebral Osteoarthritis	4 (9.8%)	5 (6.6%)	0.718
Knee Osteoarthritis	2 (4.9%)	7 (9.2%)	0.491
Constipation	19 (46%)	33 (43%)	0.762
Calcium intake	516 ± 266 (534)	737 ± 362 (702)	**0.004**
Low appetite	11 (27%)	6 (7.9%)	**0.006**
Actual smoker	1 (2.4%)	2 (2.6%)	>0.999
Actual mild to moderate alcohol user	3 (7.3%)	13 (17%)	0.142
Depression			0.188
Yes	15 (37%)	19 (25%)	
No	26 (63%)	57 (75%)	
Number of medicines	5.46 ± 2.29 (5.00)	5.21 ± 2.31 (5.00)	0.605
Benzodiazepines use	5 (12%)	7 (9.2%)	0.751
Antidepressants use	18 (44%)	25 (33%)	0.239
Antihypertensives use	12 (29%)	27 (36%)	0.493
Anticholinesterase use	2 (4.9%)	3 (3.9%)	>0.999
Antidiabetic use	3 (7.3%)	6 (7.9%)	>0.999
Measurements related to PD			
Schwab-England score	85 ± 10 (90)	84 ± 12 (90)	0.801
Hoehn Yahr			0.932
1–2	10 (24%)	18 (24%)	
2.5–3	31 (76%)	58 (76%)	
UPDRS Part III score	43 ± 16 (40)	43 ± 14 (43)	0.916
UPDRS 3.9 Standing from a chair	1.61 ± 0.74 (2.00)	1.64 ± 0.76 (2.00)	0.791
UPDRS 3.10 Gait	2.56 ± 0.63 (3.00)	2.47 ± 0.60 (2.50)	0.550
UPDRS 3.12 Postural stability	2.49 ± 1.23 (2.00)	2.39 ± 1.07 (2.00)	0.819
UPDRS 3.13 Posture	2.66 ± 1.06 (3.00)	2.66 ± 1.08 (2.00)	0.950
PIGD	9.32 ± 2.77 (9.00)	9.17 ± 2.47 (9.50)	0.943
Dyskinesia			0.608
Present	22 (54%)	37 (49%)	
Absent	19 (46%)	39 (51%)	
Visual hallucinations			0.315
Present	6 (15%)	17 (22%)	
Absent	35 (85%)	59 (78%)	
Disease duration	10 ± 6 (9)	10 ± 6 (9)	0.691
Levodopa equivalent dose	756 ± 324 (800)	724 ± 342 (675)	0.710
GDS	5.68 ± 3.45 (5.00)	4.64 ± 3.37 (4.00)	0.080
MMSE	23.8 ± 3.9 (25.0)	24.2 ± 4.3 (25.5)	0.413

PD, Parkinson Disease; UPDRS, Unified Parkinson’s disease Rating Scale; PIGD, Postural Instability and Gait Disorder; GDS, Geriatric Depression Scale; MMSE. Mini Mental Status Exam.

1 *n* (%); Average ± Standard deviation (Median).

2 Chi-square test of independence; Wilcoxon rank-sum test; Fisher’s exact test. Bold values for *p* < 0.05.

Table 4 presents the bivariate analysis results for physical performance and body composition variables. Significant associations with low protein intake included higher SARC-F scores, positive sarcopenia screening, lower handgrip strength, lower appendicular and total lean mass, lower RSMI, higher fat mass index, higher arms, legs, trunk, android and gynoid fats and osteoporosis. Variables that were statistically significant in the bivariate analysis were included in the logistic regression model. Positive sarcopenia screening and higher fat mass index were independently associated with low protein intake in the final model, as shown in Table 5. We excluded SARC-F total score, lower appendicular and total lean mass and the segmentary fat mass due to high multicollinearity based in the Variance Inflation Factor (VIF).

**Table 4:** Bivariate analysis of physical performance, body composition and protein intake.

Variables	Protein intake		*p* value^2^
	Low, *N* = 41^1^	Normal, *N* = 76^1^	
Measurements related to physical			
One or more falls in last 6 months			0.727
Yes	17 (41%)	29 (38%)	
No	24 (59%)	47 (62%)	
Two or more falls in last 6 months			0.265
Yes	8 (20%)	22 (29%)	
No	33 (80%)	54 (71%)	
Gait speed	1.29 ± 0.49 (1.27)	1.43 ± 0.53 (1.48)	0.152
SARC-F score	4.66 ± 2.72 (5.00)	3.60 ± 2.69 (3.00)	**0.041**
Positive sarcopenia screening	28 (68%)	31 (41%)	**0.005**
Handgrip strength	24 ± 9 (22)	31 ± 10 (32)	**0.001**
Low handgrip strength	7 (18%)	16 (21%)	0.625
Total SPPB score	8.56 ± 2.76 (9.00)	9.03 ± 2.51 (9.00)	0.528
Physical activity at least 3x/week for 30			>0.999
Yes	11 (73%)	27 (75%)	
No	4 (27%)	9 (25%)	
Measurements related to body composition			
Appendicular lean mass	16.3 ± 3.6 (16.4)	19.9 ± 4.8 (19.8)	**<0.001**
Total lean mass	38 ± 7 (36)	45 ± 9 (46)	**<0.001**
Confirmed sarcopenia	3 (7.3%)	9 (12%)	0.537
RSMI	6.66 ± 1.00 (6.61)	7.61 ± 1.19 (7.55)	**<0.001**
Low RSMI	8 (20%)	15 (20%)	0.973
FMI	9.8 ± 3.2 (10.0)	7.8 ± 3.5 (7.7)	**0.002**
FMI Classification			0.393
High	28 (70%)	43 (57%)	
Low	1 (2.5%)	5 (6.6%)	
Normal	11 (28%)	28 (37%)	
BMI	26.2 ± 4.1 (26.5)	26.2 ± 4.6 (26.4)	0.696
Low BMI	6 (15%)	12 (16%)	0.869
VAT volume	905 ± 629 (719)	928 ± 752 (703)	0.863
Arms fat %	37 ± 9 (38)	27 ± 9 (26)	**<0.001**
Legs fat %	36 ± 8 (37)	28 ± 9 (27)	**<0.001**
Trunk fat %	38 ± 10 (40)	31 ± 11 (33)	**0.003**
Android fat %	39 ± 12 (41)	32 ± 13 (34)	**<0.001**
Gynoid fat %	40 ± 10 (42)	31 ± 11 (31)	**0.039**
Osteoporosis WHO criteria	18 (45%)	18 (24%)	
Osteopenia	16 (40%)	35 (46%)	
Normal	6 (15%)	23 (30%)	
Calf circumference	33.4 ± 3.8 (33.5)	33.6 ± 3.6 (33.5)	0.972
Low calf circumference	10 (24%)	16 (22%)	0.763

SARC-F, Simple questionnaire to rapidly diagnose sarcopenia; SPPB, Short Physical Performance Battery; RSMI, Relative Skeletal Muscle. BMI, Body Mass Index; VAT, Visceral Adipose Tissue; WHO, World Health Organization.

1 *n* (%); Average ± Standard deviation (Median).

2 Chi-square test of independence; Wilcoxon rank-sum test; Fisher’s exact test. Bold values for *p* < 0.05.

**Table 5:** Multivariate analysis of protein intake.

Variables	OR^1^	95% CI^1^	*p* value	GVIF^1^	Adjusted GVIF^1^^,^^2^
Sex				2.9	1.7
Male	—	—			
Female	1.45	0.29, 7.58	0.650		
Positive sarcopenia screening	3.56	1.32, 10.3	**0.015**	1.1	1.1
Handgrip strength	1.05	0.97, 1.14	0.280	2.9	1.7
Low RSMI	0.37	0.15, 0.82	**0.019**	3.8	2.0
Osteoporosis NFO Criteria				1.4	1.1
Osteoporosis	—	—			
Osteopenia	1.21	0.39, 3.92	0.739		
Normal	1.19	0.25, 5.49	0.824		
FMI	1.27	1.05, 1.56	**0.016**	1.8	1.3
Change in appetite	3.72	0.92, 17.4	0.075	1.1	1.0

1 OR = Odds Ratio, CI = Confidence Interval, GVIF = Generalized Variance Inflation Factor.

2 GVIF^[1/(2*df)]. Bold values for *p* < 0.05.

The results concerning the Spearman correlation analyses between protein intake and clinical, anthropometric, and body composition variables are presented in Table 6. Fat mass index (rho −0.29; *p* 0.002) and Geriatric Depression Scale score (rho −0.21; *p* 0.021) showed a statistically significant inverse correlation with protein intake. Conversely, gait speed (rho 0.19; *p* 0.042), handgrip strength (rho 0.31; *p* < 0.001), and appendicular skeletal muscle mass index (rho 0.29; *p* 0.002) exhibited a statistically significant direct correlation with protein intake.

**Table 6:** Correlation of protein intake and clinical variables.

Parameter 1	Parameter 2	Rho	*p* value
Protein	Levodopa equivalent dose	0.09	0.369
Protein	FMI	−0.29	0.002
Protein	Calf circumference	−0.06	0.535
Protein	UPDRS Part III score	0.03	0.710
Protein	UPDRS 3.9 Standing from a chair	0.08	0.420
Protein	UPDRS 3.10 Gait	−0.05	0.595
Protein	UPDRS 3.12 Postural stability	0.00	0.967
Protein	UPDRS 3.13 Posture	0.03	0.744
Protein	Disease duration	0.04	0.659
Protein	Schwab-England score	−0.06	0.522
Protein	MMSE	0.09	0.363
Protein	Gait speed	0.19	0.042
Protein	BMI	−0.06	0.496
Protein	Handgrip strength	0.31	<0.001*
Protein	SPPB	0.10	0.304
Protein	RSMI	0.29	0.002
Protein	SARC-F	−0.05	0.631
Protein	GDS	−0.21	0.021

FMI, Fat Mass Index; UPDRS, Unified Parkinson’s disease Rating Scale; MMSE, Mini Mental Status Exam; BMI, Body Mass Index; SPPB, Short Physical Performance Battery; RSMI, Relative Skeletal Muscle Index; GDS, Geriatric Depression Scale.

Besides the concerningly high prevalence of deficient protein intake in this population, the data point toward a significant association with female sex, which may suggest sex-specific dietary patterns or metabolic differences that warrant further exploration. Additionally, the correlation with reduced apetite and low calcium intake underscores potential nutritional deficits in this population, which could exacerbate disease progression or contribute to other health issues. The link between low protein intake and several variables related to sarcopenia highlights the critical role of dietary protein in muscle preservation and function. Furthermore, our results demonstrate an association between low protein intake and unfavorable changes in body composition, potentially reflecting metabolic alterations or lifestyle factors that influence nutritional habits.

## 4 Discussion

This study aimed to estimate the prevalence of normal and low protein intake in mild to moderate PD patients, which were, respectively, 65% (*n* = 76) and 35% (*n* = 41). Clinical, parkinsonian and body composition features of the study sample were also assessed. Low protein intake was independently associated with positive screening for sarcopenia using SARC-F, higher fat mass index and low RSMI.

The average protein intake in our sample was 1.19 ± 0.41 g/kg/day. There are few studies on protein intake in PD, and the prescription of proteins in PD is also controversial, making studies on this subject relevant (22–24). Barrichela et al. conducted an extensive survey examining the dietary habits of individuals with PD. They inter-viewed 600 PD patients (53.8% male) and 600 control subjects (69% from the community) from various regions of Italy. The study found that PD patients had lower body weight and BMI compared to the control group, although their abdominal fat levels were similar. Interestingly, despite having lower energy expenditure and total daily energy expenditure, PD patients had higher calorie intake. Moreover, their total intake of calories, macro-nutrients, and micronutrients were also higher than that of the controls. The authors also described the average daily protein intake of 1.0 g/kg in the control group compared to 1.2 g/kg in PD patients (*p* < 0.001), which were like our results. Another study performed by Marczewska et al. pointed to average protein consumption by the Parkinsonian population studied (23) was 1.2 g/kg/day. Morais et al. (25) and do Carmo and Ferreira (26) found the average intake of 1.0 g/kg/day, and 1.4 g/kg/day, respectively. As levodopa and amino acids both use the large-neutral amino acid transporter for absorption in the small intestine and at the blood–brain barrier, timing and amount of dietary protein intake are critical in this condition. Levodopa should be taken 30 min before or 1 h after meals to avoid competition and decreased absorption (27). Barichella et al. and other authors suggest a low protein-diet (up to 0.8 g/kg/day) (28–30) or a protein redistribution diet (31–33) such as eating the main protein meal in the evening to make levodopa more effective and reduce motor fluctuations. Most of studies on the interaction between food and medications, particularly for levodopa, were conducted more than 20 years ago (in the 1970s, 1980s, or 1990s) and with poor methodological quality (often non-randomized or without a control group), indicating the need for more studies with better methodology (34).

In older adults, PROT-AGE study group recommends average daily intake of protein in the range of 1.0 to 1.2 g/kg/day (35). ESPEN guideline also suggests protein daily amounts of 1.0–1.2 g/kg/day (19). Both recommendations are related to healthy older adults. According to PROT-AGE study group most older persons with acute or chronic diseases require higher dietary protein (1.2–1.5 g/kg BW/d); but people with severe sickness or injury or noticeable malnutrition may require up to 2.0 g/kg/day (35).

A study conducted in Brazil at the University of São Paulo with 295 older adults, with a mean age of 70.41 ± 7.48 years and a higher proportion of women (81.69%) compared to men (18.31%), found a prevalence of 69.15% of older adults with low protein intake (considered as less than 1 g/kg/day for those who are eutrophic and overweight and less than 1.2 g/kg/day for those who are undernourished) (36). The National Dietary Survey 2008–2009, involving a total of 4,286 Brazilian elderly individuals (aged 60–104 years), revealed the average protein intake of 75.5 grams. Women had a significantly lower average protein intake compared to men (83.6 grams versus 68.3 grams; *p* < 0.001). The northern region showed the highest protein-energy percentage (21.5%; 95% CI, *p* < 0.05), while the southern region had the lowest (17.9%; *p* < 0.01). The protein-energy percentage was greater in rural areas compared to urban areas (20.2% versus 19.8%; *p* < 0.05) (37).

A higher proportion of men had adequate protein intake comparing to women in the present study. Body composition varies between men and women, with women having proportionally greater fat mass and men having more muscle mass (38). Sex steroids can modulate disparities in body composition. For instance, reduced estrogen levels, such as those encountered during menopause, have been associated to a predilection for visceral adipose tissue (VAT) accumulation and an increased cardiometabolic risk (39). Additionally, decreasing testosterone levels in men can lead to increased visceral fat (40). Furthermore, new genome-wide association studies have identified genetic markers unique to each sex that promote fat formation (41). According to Bennett (42), women consume an average amount of 78 grams/day of protein, while men consume 86.9 grams/day. However, when adjusted for body weight, women’s protein consumption surpassed that of men (1.13 g/kg/day × 1.04 g/kg/day) (42). Regarding body composition, according to Schorr (38), men have a higher amount of muscle mass than women, resulting in an increased need for a higher protein intake (38).

Low protein intake was significantly associated with low handgrip strength in PD patients in the present study. Also, gait speed, handgrip strength and appendicular skeletal muscle mass index exhibited a direct correlation with protein intake. Several studies have also described that lower intake of protein has been linked with lower muscular strength (43–45). Indeed, these findings confirm how essential it is to consume enough protein to maintain muscle strength, especially for populations as older people and those with chronic diseases (44, 46, 47). However, caution is needed with these findings. It is essential to understand that protein intake alone may not fully explain changes in muscular strength, since other factors such as physical activity levels, total food quality, and genetic predispositions all play significant parts (48). The higher the total protein intake up to 1.5 g/kg combined with resistance training, the better effect in muscle strength according to a recent systematic review and meta-analysis (49).

We found an association between low protein intake and higher fat mass index. Protein plays a fundamental role in managing weight and body composition. Diets with a higher amount of protein favor the maintenance or increase of lean mass, in addition to contributing to a reduction in caloric intake (50). This caloric reduction is possibly due to the increase in satiety induced by this macronutrient, an effect that may be related to the stimulation of the secretion of gastrointestinal hormones, such as cholecystokinin and glucagon-like peptide-1 (GLP-1) (51, 52). A higher protein intake is associated with reduced total body fat, including the abdominal region, especially in overweight individuals who practice physical exercise (53–55). Although there is no consensus on the amount of protein needed to promote body fat reduction, evidence suggests that diets with protein intake equal to or greater than 25% of total daily caloric intake or ≥1 g/kg/day show benefits during the process of weight loss in older individuals, preserving lean mass and reducing body fat (56). Weight loss in PD is frequently reported and has been associated especially with the severity of the disease, however, an increase in BMI and a redistribution of body composition, characterized by an increase in body fat and a reduction in muscle mass, have also been shown concomitantly, associated with decreased protein intake (57). However, it is important to highlight that body composition is influenced by several factors in addition to protein intake, such as caloric intake, level and type of physical exercise, genetic aspects, associated diseases, and the use of medications, demonstrating the complexity of mechanisms that regulate body composition (58–61).

Reduced appetite was associated with lower protein intake. Various factors may contribute to reduced appetite in PD, including “inflammaging” (62, 63), dysautonomic symptoms (such as constipation and dyspepsia) (64–66), dysphagia (67), antiparkinsonian side effects (66, 68, 69), depression (65, 69, 70) and dementia syndrome (71, 72). Additionally, these issues are exacerbated by age-related sarcopenia, which can result from inadequate nutrition, physical inactivity, and endocrine dysfunctions, contributing to a negative cycle of reduced appetite and consequently lower protein intake (73–76).

The study also showed association between low protein intake and lean appendicular mass index. Several studies already showed that dietary protein plays a crucial role as it is a modifiable factor that influences the growth and maintenance of skeletal muscle (10, 14, 48–53). A protein intake level of 1 to 1.2 g/kg/day for elderly individuals and those with chronic diseases, along with the consumption of 10 to 15 grams of essential amino acids (containing more than 3 grams of leucine), which corresponds to approximately 25 to 30 grams of high biological value protein in each of the three main meals, is recommended to improve protein synthesis (49, 51, 56, 77).

In the present study, sarcopenia identified by a positive SARC-F questionnaire was independently associated with low protein intake. Sarcopenia and PD are both conditions associated with aging that might have a shared underlying mechanism (78). The SARC-F questionnaire includes five questions that assess hand strength for lifting a 5 kg weight, thigh strength for standing up from a chair, strength and balance for walking between rooms and climbing 10 flights of stairs, and a history of falls (79). The items of the SARC-F are linked to functional performance, and functional performance is associated with adequate protein intake. The association between the SARC-F and protein intake can be explained by the well-established relationship between adequate protein intake and muscle strength (14, 44, 49). Elevated SARC-F scores were strongly correlated with disease severity and dependency in both activities of daily living (ADL) and instrumental activities of daily living (IADL) in the present study. It has been suggested that the SARC-F is more sensitive in detecting muscle function compared to muscle mass (80). Thus, SARC-F may be a tool to assess PD patients for the presence and functional decline.

This study has several limitations. First, it involves a small sample of patients with mild to moderate PD according to HY (1 to 3). Therefore, we did not include patients with severe disease who exhibit more malnutrition, dysphagia, cognitive alterations, dyskinesias, and consequently more sarcopenia. We cannot, therefore, extrapolate these results to all patients with PD. Additionally, the collection of the inventory of protein and calcium consumption was conducted through questions about intake over the last 24 h, which means the values found may be underestimated or overestimated since the information was collected only once. A three-day food intake record is considered a more accurate evaluation method than food frequency questionnaires and 24-h recall, as it allows participants to directly record their food intake and avoid memory bias. The prevalence of sarcopenia varies due to the use of different definitions and diagnostic tools for sarcopenia, as well as patient selection criteria, such as the inclusion of patients with more advanced PD (HY 4–5). Additionally, there are different ways to measure body composition and different diagnostic criteria. All these methodological differences create limitations for comparing our results. The sarcopenia cut-off points used in this study were derived from those recommended by EWGSOP 2 for healthy adults, as there are no defined cut-off points for patients with PD. Moreover, there are currently no validated methods for diagnosing dynapenia by assessing lower limb strength in PD patients. Implementing a validated method to measure lower limb strength is likely to enhance the accuracy of sarcopenia detection in PD patients.

The association between low protein intake and the increased risk of sarcopenia, as well as the reduction in muscle strength in patients with Parkinson’s disease (PD), highlights the importance of regular nutritional monitoring in the management of PD and the prevention of sarcopenia. It is essential to monitor dietary intake, with an emphasis on protein consumption, while also considering other nutritional aspects, such as daily caloric intake, overall diet quality, and related factors. This comprehensive monitoring contributes to the preservation of muscle strength, improved functionality, and the promotion of quality of life for patients. Future research directions include evaluating the impact of dietary changes over time on the progression of PD and complications such as sarcopenia and dynapenia. Studies on the effects of protein supplementation are also necessary, as well as investigations into how nutritional interventions interact with other clinical and lifestyle factors. Additionally, the implementation of technologies, such as dietary tracking apps, can be a valuable tool for conducting more frequent and accurate assessments, optimizing nutritional monitoring and clinical care for these patients.

Our study revealed a significant prevalence of low protein intake among PD patients at HY stages 1–3, based on the ESPEN recommendation (2022) of at least 1 g/kg/day of protein intake. This low intake was independently associated with positive sarcopenia screening through SARC-F, reduced lean appendicular mass, and a high fat mass index. A deeper understanding of the relationship between protein intake and body composition in PD may enhance long-term outcomes for patients. We suggest that healthcare providers inquire about both the quality and quantity of nutrition, as this population is at higher risk for sarcopenia and malnutrition.

## Data Availability

The raw data supporting the conclusions of this article will be made available by the authors, without undue reservation.
